# Editorial: Synovial pathobiology and pathogenesis of inflammatory arthritis, volume II

**DOI:** 10.3389/fmed.2024.1462668

**Published:** 2024-07-25

**Authors:** Annabelle Small, Frances Humby, Mihir D. Wechalekar

**Affiliations:** ^1^College of Medicine and Public Health, Flinders University, Adelaide, SA, Australia; ^2^Department of Rheumatology, Guy's and St Thomas' National Health Service (NHS) Foundation Trust, London, United Kingdom; ^3^Department of Rheumatology, Flinders Medical Centre, Adelaide, SA, Australia

**Keywords:** synovium, rheumatoid arthritis, psoriatic arthritis, post-traumatic knee osteoarthritis, pathogenesis, precision medicine

The development of predictive tools for the prognosis of inflammatory arthritis is currently a key area of research in rheumatology. In our previous Research Topic (1), we explored current ongoing endeavors seeking to find these tools and future strategies that may be adopted to make these much-needed predictions. In the second volume of *Synovial pathobiology and pathogenesis of inflammatory arthritis*, we now delve deeper into the underlying rheumatoid arthritis (RA) and psoriatic arthritis (PsA) synovial tissue, explore the role of inflammation in post-traumatic osteoarthritis (PTOA), investigate synovial myeloid cell subsets and their role in remission of RA, and finally, explore the link between disease activity indices with disease progression.

The presentation of RA can be remarkably heterogeneous. Genetics, exposure to external factors, and microbiota are among many factors that can influence disease course, however these, either alone or paired with clinical parameters, are insufficient for predicting prognosis and treatment outcomes. Recently, a major area of research interest for the development of precision medicine in RA management is within the synovial tissue itself. The clinical heterogeneity of RA is reflected in heterogeneous inflammatory infiltrates which can vary enormously between patients, and this Research Topic is neatly reviewed by Bykerk. This contribution summarizes the establishment of the now accepted concept of histological synovial pathotypes, how these are determined and the relationship of these pathotypes with clinical measures. Advances in approaches for ST molecular and cellular phenotyping are discussed, and current associations between synovial pathobiology and clinical observations summarized. Collaborative, combined approaches utilizing clinical data alongside multi-omic strategies may unravel the importance of bona fide RA “pathotypes,” and may together allow the development of personalized approaches capable of effectively treating disease in the early stages.

Like RA, the clinical presentation of PsA can vary enormously, and the inflamed PsA synovial tissue can share numerous general features with that of RA, making diagnosis oftentimes challenging. Additionally, as worldwide interest in the utility of synovial tissue analyses in unraveling pathobiology increases, it is important to understand the similarities and differences between synovial histopathologic findings in different inflammatory conditions. To this end, Tenazinha et al. present a thorough narrative review of PsA histopathology, where they highlight the fundamental differences and similarities between the PsA and RA synovium and outline the clear unmet need for synovium-specific biomarkers in PsA. With respect to inflammatory infiltrates, total counts of T cells and plasma cells are typically lower in PsA compared to RA, while the proportion of these and mast cells are higher. Meanwhile, lymphoid follicles [prevalence of ~50% (2)] and fibrosis are commonly observed in PsA along with marked synovial vascularity. While the Th-17 pathway is undoubtedly important in PsA—shown by the therapeutic efficacy of anti-IL-17 therapies—synovial IL-17 and IL-22 do not allow differentiation from RA tissue. However, a more useful marker for distinguishing between conditions may potentially be IL-35, which is significantly reduced in PsA compared to RA.

PTOA occurs in anywhere from 25 to 50% of patients following major trauma. While the initial insult and subsequent mechanical processes have long been though to drive PTOA, evidence suggests a role of hemarthrosis and inflammation in PTOA progression, which are reviewed succinctly in the contribution by Evers et al. The group provides a brief, but comprehensive introduction of the relevant knee anatomical structures involved in PTOA, along with discussion of the incidence, diagnosis, current therapies and prognosis for patients with knee injuries. They summarize how hemarthrosis, or articular bleeding, has been linked to PTOA development through the process of erythrocyte breakdown, which results in hemosiderin deposit accumulation in synovial macrophages and synoviocytes, altering their pro-inflammatory function. Indeed, following knee injury, synovitis is often observed within the synovium and in both human and animal studies, this synovitis has been directly linked to the injury itself. Consequently, because of the link between inflammation and PTOA, emerging studies on the utility of anti-inflammatory therapies in the progression of PTOA in humans have been conducted. Meanwhile, in experimental models, anti-inflammatory cell-based therapies have been conducted and have also shown promising results. However, despite promising patient-reported outcomes in early human trials, and despite promising outcomes in murine models, there remains an enormous amount of space in this area for further clinical studies. Such studies, paired with an increase in our understanding of the role of inflammation in PTOA pathogenesis, are needed to enable the development of effective therapies for patients with, and those at risk of developing PTOA, and is likely to form an exciting area of OA research in the future.

Myeloid populations are a critical constituent of the healthy joint, where they constantly survey their microenvironment and work to maintain homeostasis. In RA, there a is marked increase in monocytes and macrophages, and while these undoubtedly contribute to local inflammation, subpopulations associated with remission have been described (3). Mining data from four previously published single-cell (sc)RNA-sequencing datasets, along with their own generated dataset, Hu et al. describe a subpopulation of COL3A1^+^ macrophages with an M2-like, anti-inflammatory phenotype. Pseudotime analysis of the identified COL3A1^+^ population suggested that these may arise from macrophage-myofibroblast transition, while functional work demonstrated that culture in the presence of TGF-β1 induced COL3A1 expression and collagen production in THP-1 derived macrophages, and decreased inflammatory cytokine production. Additionally, COL3A1^+^ THP-1 macrophages exhibited increased adhesion in *in vitro* adhesion assays, and formed a membrane-like structure observed by immunofluorescence following culture onto glass slides. Taking these observations together, Evers et al. suggest a role for COL3A1^+^ macrophages in the formation of the physical and immunological synovial lining layer, and a potential role for these cells in remission. Considering increased recent interest in myeloid and fibroid populations in the pathogenesis of RA, further study in this are likely to reveal whether this subset may be targetable and pose a future therapeutic approach.

Despite our growing understanding of synovial pathotypes and heterogeneity, the relationship between these and disease activity measures remains unclear. Rodriguez-García et al. sought to compare the capacity of the “Hospital Universitario La Princesa Index” (HUPI), DAS28-ESR, and SDAI with changes in physical function (measured by HAQ), inflammation (measure by IL-6 serum levels), and radiologic progressions. Statistical comparisons indicated that HUPI was comparable with DAS28-ESR and SDAI as an explanatory variable for HAQ, IL-6 levels, and radiological progressions. HUPI best explained decline in HAQ, and was the second best explaining IL-6 levels, while none of the measures correlated well will radiological progressions, together supporting the use of HUPI for research correlations.

Altogether, the body of work presented in this volume paints a comprehensive picture of synovial tissue heterogeneity and highlights the importance of unraveling the underlying pathobiological processes of synovitis within the context of clinical measures. Only with this integrated insight can we bridge the gap between clinical examination and basic science findings, and together improve outcomes for patients affected by inflammatory arthritis.

## Author contributions

AS: Conceptualization, Writing – original draft. FH: Conceptualization, Methodology, Project administration, Writing – review & editing. MW: Conceptualization, Methodology, Project administration, Supervision, Writing – review & editing.
